# Developing a Protein-Interactions Ontology

**DOI:** 10.1002/cfg.244

**Published:** 2003-02

**Authors:** Esther Ratsch, Jörg Schultz, Jasmin Saric, Philipp Cimiano Lavin, Ulrike Wittig, Uwe Reyle, Isabel Rojas

**Affiliations:** 1 European Media Laboratory (EML) Schloss-Wolfsbrunnen-Weg 31c Heidelberg 69118 Germany; 2 Institut für Maschinelle Sprachverarbeitung (IMS) University of Stuttgart Stuttgart Germany; 3 Max-Planck-Institute for Molecular Genetics Berlin Germany


**Comparative and Functional Genomics**
*Comp Funct Genom* 2003; **4**: 85–89.
Published online in Wiley InterScience (www.interscience.wiley.com). **DOI:** 10.1002/cfg.244

**Conference Review**

### Developing a protein-interactions ontology

Esther Ratsch[1]*, Jörg Schultz[3], Jasmin Saric[1], Philipp Cimiano Lavin[1,2], Ulrike Wittig[1], Uwe Reyle[2] and Isabel Rojas[1]

[1] *European Media Laboratory (EML), Heidelberg, Germany*
[2] *Institut für Maschinelle Sprachverarbeitung (IMS), University of Stuttgart, Stuttgart, Germany*
[3] *Max-Planck-Institute for Molecular Genetics, Berlin, Germany*

**Correspondence to:*
*Esther Ratsch, European Media Laboratory,*
*Schloss-Wolfsbrunnen-Weg 31c,*
*69118 Heidelberg, Germany.*
*E-mail: Esther.Ratsch@EML.Villa-Bosch.de*

Received: 15 November 2002
Revised: 26 November 2002

**Keywords:** **ontology; signal transduction; protein interaction**

### Why a protein-interactions ontology?

The prediction and analysis of a protein's function is an ongoing challenge in the field of genomics. With upcoming datasets on protein interactions [9], it is becoming evident that the function of a protein can only be understood when taking its interaction with other molecules into account. Most current approaches to the classification and description of protein function, such as the Gene Ontology [8], focus on single proteins. These annotation efforts should be paralleled by the development of ontologies dealing with the interactions of a protein with other biomolecules. Currently, most approaches to building such ontologies focus on metabolism [3,6]. So far, for interactions, only high-level classifications have been created [4], developed to assist information extraction from text. In addition to assisting text mining, a more fine-grained (in comparison to these classifications) ontology on protein interactions could be helpful in database development and information mining. As an ontology captures domain knowledge in a computer-understandable way, it can be used for inferencing, i.e. deriving new knowledge from existing data.

There are two important points to consider in developing such a formal ontology: (a) it should be independent of its final use; and (b) it should not only restrict itself to a controlled vocabulary but the concepts should be related to each other in a semantically consistent manner, and rules governing these definitions and relations should be incorporated whenever necessary. Here we describe our approach for developing such an ontology.

### Development of the ontology

Our general procedure when developing an ontology involves five steps:

1. The identification of its scope.
2. The identification of the concepts needed and the properties thereof.
3. The decision on how these entities and relationships are to be represented.
4. The definition of rules and constraints.
5. The formalization.

Copyright © 2003 John Wiley & Sons, Ltd.

### Scope

The ontology we are developing is intended to represent interactions between proteins and other cellular compounds, including proteins, nucleic acids, lipids and ions. We have restricted the description of these interactions to a molecular level, i.e. to the level of interactions with amino acids. Although it would be feasible to describe the interactions at an atomic level, based on three-dimensional structures, most of the concepts can be sufficiently described at the higher level of amino acids, and most of the interaction data available is at that level.

In the initial phase, we focus on interactions associated with signal transduction, for two reasons. First, these processes rely strongly on protein interactions. Second, they are important regulatory processes, frequently involved in the development of diseases. We decided to concentrate on the representation of qualitative aspects of these pathways. No quantitative properties, such as the concentration of compounds involved, are modelled, given that, in contrast to metabolic pathways, the concentration of compounds involved in signal transduction pathways is often not measurable.

A signal transduction pathway contains at least parts of a path that, in most cases, transfers a signal from the outside of the cell into its nucleus. This signal transfer is performed by a chain of interacting proteins, as we will exemplify with the Jak–Stat pathway (Figure 1; for a review see [1]). It starts at the cellular membrane, where a ligand binds to a receptor, leading to the oligomerization of the receptor. The Jak proteins bound to the intracellular part of the receptor are activated upon oligomerization of the receptor. They phosphorylate both the receptor and Stat proteins, which bind to the phosphorylated receptor. Once phosphorylated, Stat proteins can dimerize, which enables them to enter the nucleus and upregulate the transcription of a set of target genes.

Such signal transduction cascades enable the cell to respond to environmental changes. Since several signal transduction pathways are interrelated and form a regulatory network, fast and appropriate answers to such changes are possible.

### Concepts involved

When looking at a typical signal transduction pathway we can identify two main concepts needed for the representation of protein interactions: the interacting compounds and the interactions themselves. The interacting compounds are proteins, nucleic acids and other compounds, such as ions, and can be composed of sub-parts. Proteins, for example, are composed of amino acids and contain regions with defined functions, known as domains. Apart from the actual characteristics of a protein, such as its sequence, the molecular weight and the isoelectric point, there are at least three different factors

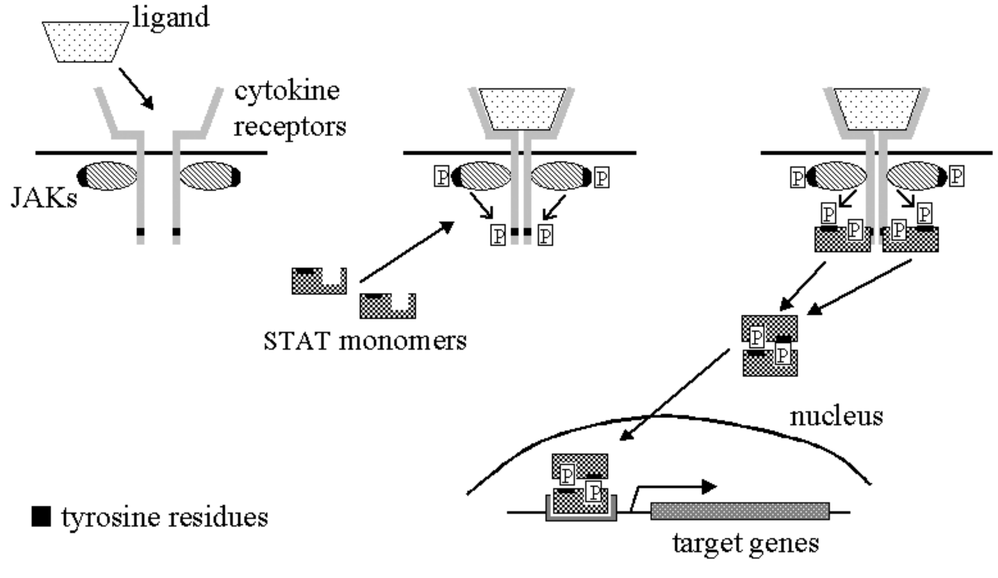

**Figure 1.** Schematic model of the Jak–Stat pathway (see text)

Copyright © 2003 John Wiley & Sons, Ltd. 

that define the interaction potential of a protein: its modifications, its location and its binding partners.

The protein interactions are what really make up a signal transduction pathway. There are different types of interactions, e.g. binding, phosphorylation, cleavage or translocation. Although from a conceptual point of view translocation, phosphorylation and cleavage do not constitute actual interactions, but are really the consequence of an interaction, in the biological domain these concepts are grouped under the heading of interactions, probably because they are integral parts of many signal transduction pathways and involve interaction with proteins. Thus, we have decided to refer to them also as interactions. An additional classification has been made based on functional similarity, grouping more than 100 verbs extracted from SWISS-PROT function descriptions into 11 not-disjoint, i.e. partially overlapping, classes: Control/Regulation; Biochemical Interactions; Logical Interactions; Bind/Dissociate; Formation; Integrity; Availability; Change of Location; Modification of Structure; Special Processes/Reactions; and Order. We are now working on the consolidation, extension and hierarchical arrangement of these classifications.

### Representation of the identified concepts

The interaction potential of a protein is defined in terms of its state. A protein state is made up of a localization, a list of modifications and a list of binding partners, e.g. a protein can be phosphorylated at one amino acid residue, be located in the cytoplasm and be bound to no other proteins. This is a similar approach to that of the LiveDIP [2].

An interaction can be represented as an event with a pre- and a post-condition. The pre-condition is defined in terms of the characteristics of the states that the participating proteins have to fulfil in order for the interaction to take place. The post-condition describes the resulting states of these proteins. Therefore, an interaction event is described by the pre-states and post-states of the involved proteins, e.g. a translocation event changes the value of the localization but leaves the other attributes unchanged, whereas a phosphorylation event changes only the modifications value.

### Rules, constraints and their formalization

The rules describing protein interactions in a computer-understandable way and the constraints included to guarantee the consistency of data are what distinguish a formal ontology from a controlled vocabulary. These rules and constraints represent the knowledge of field experts used when validating data, sometimes without even noticing. Even if these rules and their scientific significance are known to the biologists, most of them are not formalized, which is necessary for validation processes to be carried out by computers. An example of such a rule is the fact that a eukaryotic protein can only be phosphorylated at certain amino acids, which are tyrosine (Y, phosphorylated by a *Y-kinase*), or threonine (T) or serine (S) (phosphorylated by an *S/T-kinase*). These two kinases are protein kinases, which are types of proteins. This can be formalized in first-order logic in the following way:

$$\forall X : [\text{protein-kinase}(X) \rightarrow \text{protein}(X)]$$
$$\forall X : [\text{Y-kinase}(X) \rightarrow \text{protein-kinase}(X)]$$
$$\forall X : [\text{S/T-kinase}(X) \rightarrow \text{protein-kinase}(X)]$$
$$\forall X : [\text{S/T-kinase}(X) \rightarrow \neg\text{Y-kinase}(X)]$$
$$\forall X : [\text{Y-kinase}(X) \rightarrow \neg\text{S/T-kinase}(X)]$$

We decided to use first-order logic for the formalization to keep the ontology development independent of its implementation, which can be done using different tools and for different applications.

If there is anything that comes close to a *lingua franca* in logic, computer science and their applications, it is first-order predicate logic (FOL). FOL has well-understood semantics and inference methods. Furthermore, there are correspondence theories that allow one to express temporal and modal dependencies within FOL. Hence, the expressive power of FOL was sufficient for our purposes. This does not mean that we want, or need to, exploit all the capacities of FOL in modelling our domain, but it is convenient to have such a powerful language to make the domain knowledge explicit and precise. For real applications we then can translate our axioms into systems with less expressive power (e.g. description logics) but that guarantee certain computational properties (e.g. decidability).

Copyright © 2003 John Wiley & Sons, Ltd. 

To represent interactions, let us take as an example a phosphorylation, represented, like all interactions, as an event. This implies that we have to specify the pre- and post-conditions. Before the phosphorylation takes place the specific part R (*residue*) of the protein P [*is_residue_of(R,P)* and *protein(P)*], which is to be phosphorylated, has to be unmodified [$\neg$*modified(R)*]. After the phosphorylation takes place, the specific residue will be phosphorylated [*phosphorylated(R)*]. A residue is modified if it has been phosphorylated, glycosylated, etc., formally:

$$\forall \mathrm{R, s[s : modified(R)} \leftrightarrow \mathrm{(phosphorylated(R)} \vee \mathrm{glycosylated(R)} \vee \ldots)]$$

Assuming that the predicates *protein(X), protein-kinase(X), is_residue_of(X,Y) phosphorylated(X)* and *result(X)* have already been defined in the ontology, a phosphorylation can be formalized as:

$$\forall \mathrm{e, s, s', P, Q, R[e : phosphorylation(P, Q, R)} \leftrightarrow \mathrm{(protein(P)} \wedge \mathrm{protein\text{-}kinase(Q)} \wedge \mathrm{is_residue_of(R, P)} \wedge \mathrm{s} : \neg \mathrm{modified(R)} \wedge \mathrm{s'} : \mathrm{phosphorylated(R)} \wedge \mathrm{result(e)} = \mathrm{s'} \wedge \mathrm{s} \supset\subset \mathrm{e})]$$

Here, the temporal order of the states and events is imposed by the use of two relations. The function *result*, applied to an event $e$, returns the resulting state $s'$ of the event. This implies a temporal succession of $e$ by $s'$, such that $s'$ comes immediately after $e$. In addition, the abut-relation $s \supset\subset e$ indicates that $s$ is immediately followed by $e$.

Given this formalization of a phosphorylation event and the above hierarchy of protein-kinases, we can now express that the phosphorylated residue and the type of kinase are mutually dependent:

$$\forall \mathrm{e, P, Q, R[e : phosphorylation(P, Q, R)} \wedge \mathrm{S/T\text{-}kinase(Q)} \rightarrow \mathrm{(threonine(R)} \vee \mathrm{serine(R))]}$$

$$\forall \mathrm{e, P, Q, R[e : phosphorylation(P, Q, R)} \wedge \mathrm{(threonine(R)} \vee \mathrm{serine(R))} \rightarrow \mathrm{S/T\text{-}kinase(Q)]}$$

Such rules have to be included in the ontology to prevent phosphorylation events on any other amino acid. In addition, the phosphorylation event is a kind of interaction, which gives us the possibility to infer the co-location of the interacting partners:

$$\forall \mathrm{e, P, Q, R[e : phosphorylation(P, Q, R)} \rightarrow \mathrm{e : interaction(P, Q)]}$$

$$\forall \mathrm{e, P, Q[e : interaction(P, Q)} \rightarrow \mathrm{loc(Q)} = \mathrm{x} \wedge \mathrm{loc(P)} = \mathrm{y} \wedge \mathrm{at(x, y)]}$$

This formula assumes a fine-grain ontology of locations, although we are aware that most localization data available are limited to the level of compartments in which compounds are located. The inference that the interacting elements have to be co-located is limited to being close to each other [*at(x,y)*] and thus in the same compartment.

### Challenges met

In our approach to develop an ontology for protein interactions, we had to face different challenges, which mainly emerged from the need for abstraction, the actual implementation of the ontology, the domain being analysed and the interdisciplinarity of the group. This last factor turned out to be both a challenge and a benefit to our work. The group working on this ontology is composed of computer scientists, computational linguists and biologists. These different backgrounds force all group members to formulate their concepts in a precise and understandable way. As a result, ambiguities become clear and can be approached at a very early stage in ontology development. Furthermore, every discipline plans to use the ontology for different projects, leading to the development of a project independent ontology.

Challenges typical for the biological domain arise because of the granularity of information. Realistically, an interaction between two proteins takes place between defined atoms of distinct amino acids of these proteins. However, this level of detail is usually not known. For well-understood and experimentally characterized interactions, the interacting amino acids might be known, but in most cases only the interacting domains are described. In the case of data derived from large-scale proteomics analyses, only the names of the interacting proteins are given. Frequently, these different levels of detail are mixed, as in cases where

Copyright © 2003 John Wiley & Sons, Ltd. 

one partner is only known as the protein name, but for the other partner the amino acids involved are also given. In addition to this three-layered granularity of information, another granularity exists. Signal transduction pathways can be described on a general (organism-independent) level, talking about protein families, e.g. in the way the Jak–Stat pathway has been described above. However, there are several members of the Jak- and Stat-protein families, respectively, so these pathways can also be described at the level of orthologous groups, e.g. where a Jak2 protein interacts with a Stat5 protein. Finally, one could be interested in the actual proteins within a specific organism, e.g. distinguishing mouse-Jak1 from rat-Jak1. These two types of granularities have to be combined to allow the representation of partial information.

### Future developments

We are currently improving the representation of the underlying concepts and increasing the coverage of the ontology. Although it is still in a very early state, we have started to use the knowledge generated in developing the interactions ontology in related projects. To mine relations between protein interactions and metabolic pathways, the ontology is being integrated into an already existent ontology developed at the EML [5], which focuses on metabolic pathways.

Based on parts of the protein interactions ontology, we automatically translate the content of SWISS-PROT feature table lines into fully structured tree-like representations that can be queried and graphically displayed by using the TIGERSearch engine (developed at the University of Stuttgart; Lezius, PhD thesis to be submitted). The TIGERSearch software is a specialized search engine for querying datasets of tree-like structures, so-called 'treebanks'.

In view of the increasing number and coverage of databases concerned with signalling pathways, such as CSNDB [7], we envision using the ontology to annotate these pathways. This could lead to an increased insight into the molecular details of the interactions within cellular networks.

This definition of sub-projects, where the ontology plays a key role, helps us on the one hand to evaluate its applicability, and on the other hand justifies the amount of effort that goes into the process of developing an ontology. Based on our experiences, the creation of interdisciplinary groups for ontology development turned out to be very beneficial. The combination of these two factors, i.e. the multiple applications and the interdisciplinary nature of the group, is an approach we highly recommend to other groups wanting to create an ontology for a specific domain.

### Acknowledgements

We are very grateful to the Klaus Tschira Foundation (KTF) and the BMBF (Project Bioregio 12212) for their financial support.

### References

1. Aaronson DS, Horvath CM. 2002. A road map for those who know JAK–STAT. *Science* **296**(5573): 1653–1655.
2. Duan XJ, Xenarios I, Eisenberg D. 2002. Describing biological protein interactions in terms of protein states and state transitions: the LiveDIP database. *Mol Cell Proteom* **1**(2): 104–116.
3. Karp PD. 2000. An ontology for biological function based on molecular interactions. *Bioinformatics* **16**(3): 269–285.
4. Rzhetsky A, Koike T, Kalachikov S, *et al*. 2000. A knowledge model for analysis and simulation of regulatory networks. *Bioinformatics* **16**(12): 1120–1128.
5. Rojas I, Bernardi L, Ratsch E, *et al*. 2002. A database system for the analysis of biochemical pathways. *In Silico Biol* **2**(2): 75–86.
6. Schomburg I, Chang A, Schomburg D. 2002. BRENDA, enzyme data and metabolic information. *Nucleic Acids Res* **30**(1): 47–49.
7. Takai-Igarashi T, Nadaoka Y, Kaminuma T. 1998. A database for cell signaling networks. *J Comp Biol* **5**(4): 747–754.
8. The Gene Ontology Consortium. 2000. Gene ontology: tool for the unification of biology. *Nature Genet* **25**: 25–29.
9. Xenarios I, Eisenberg D. 2001. Protein interaction databases. *Curr Opin Biotechnol* **12**(4): 334–339.

Copyright © 2003 John Wiley & Sons, Ltd. 
